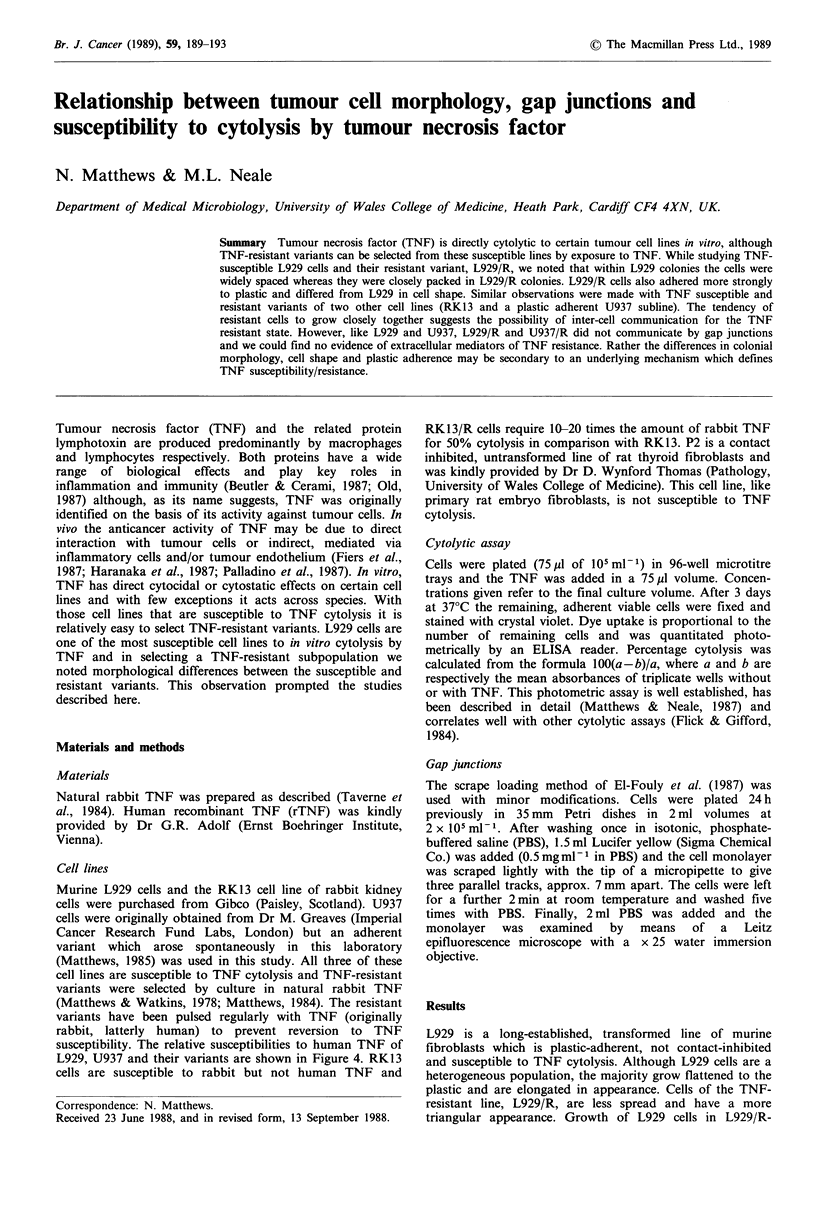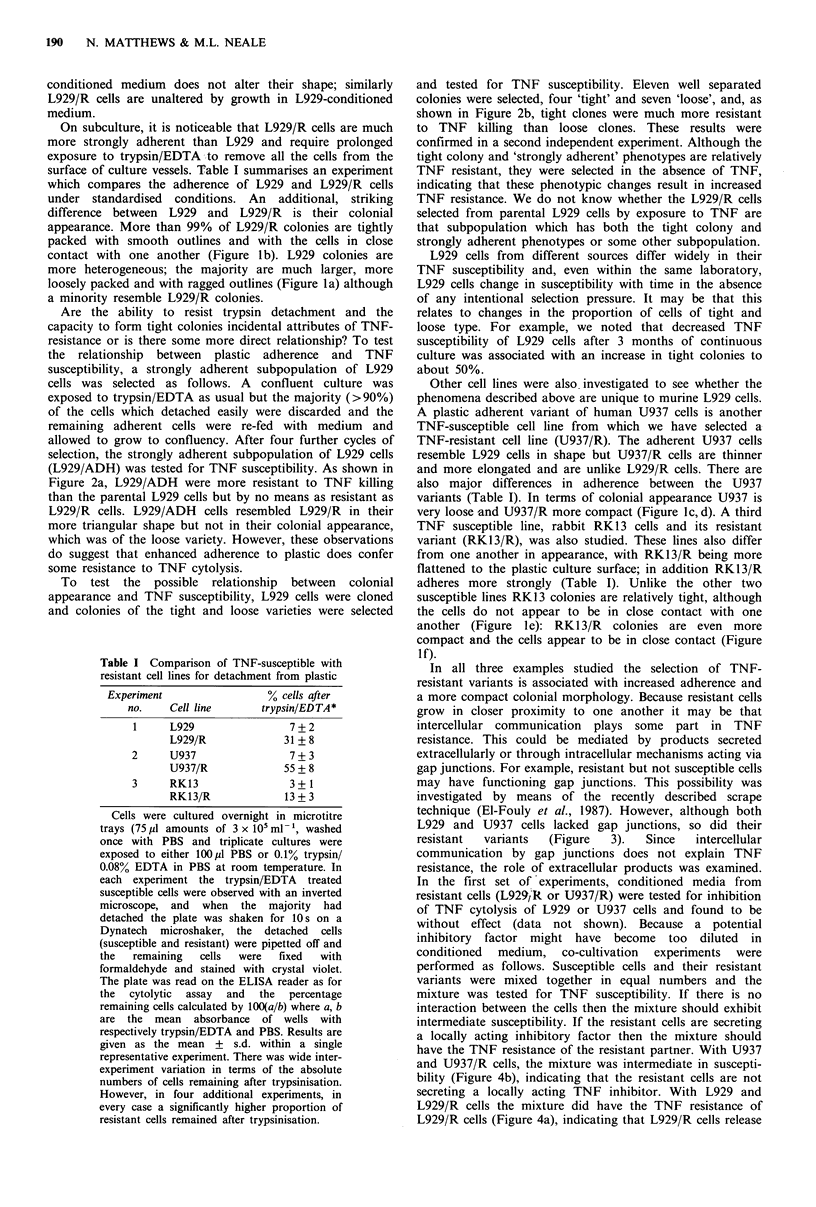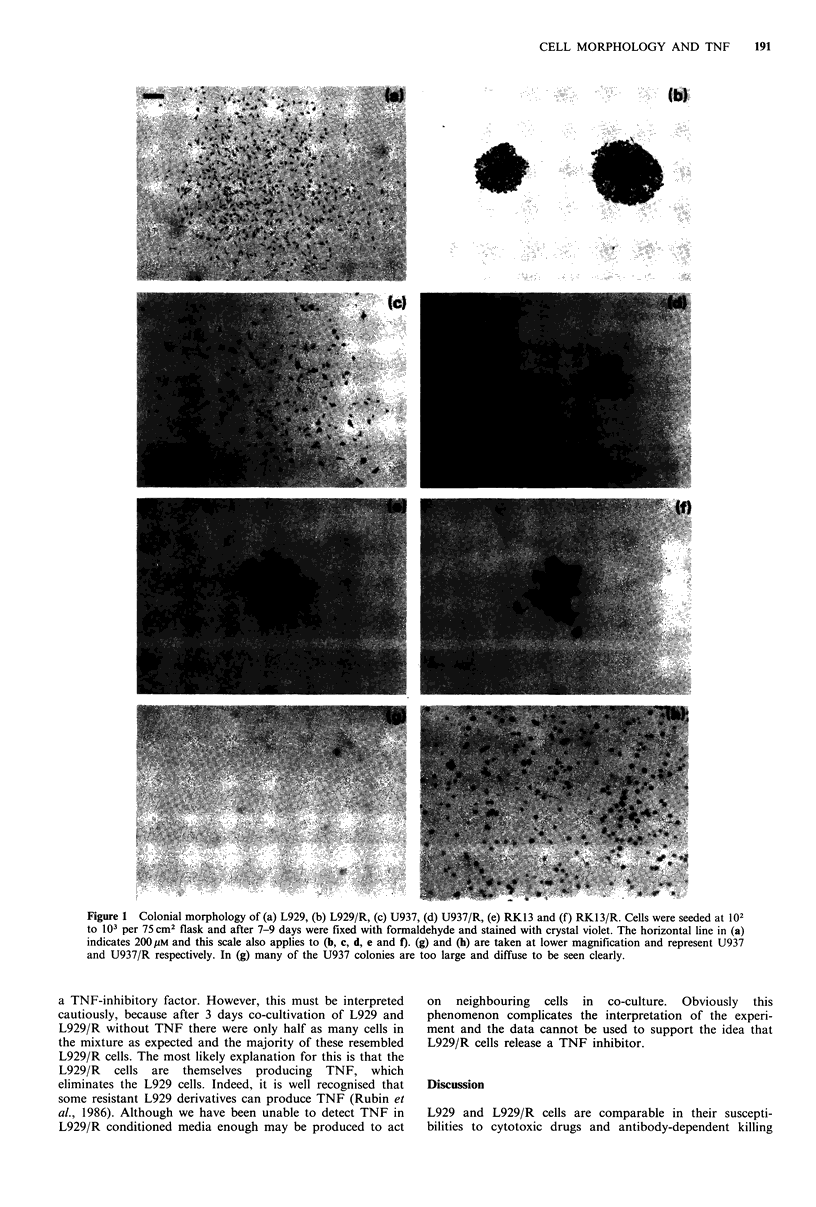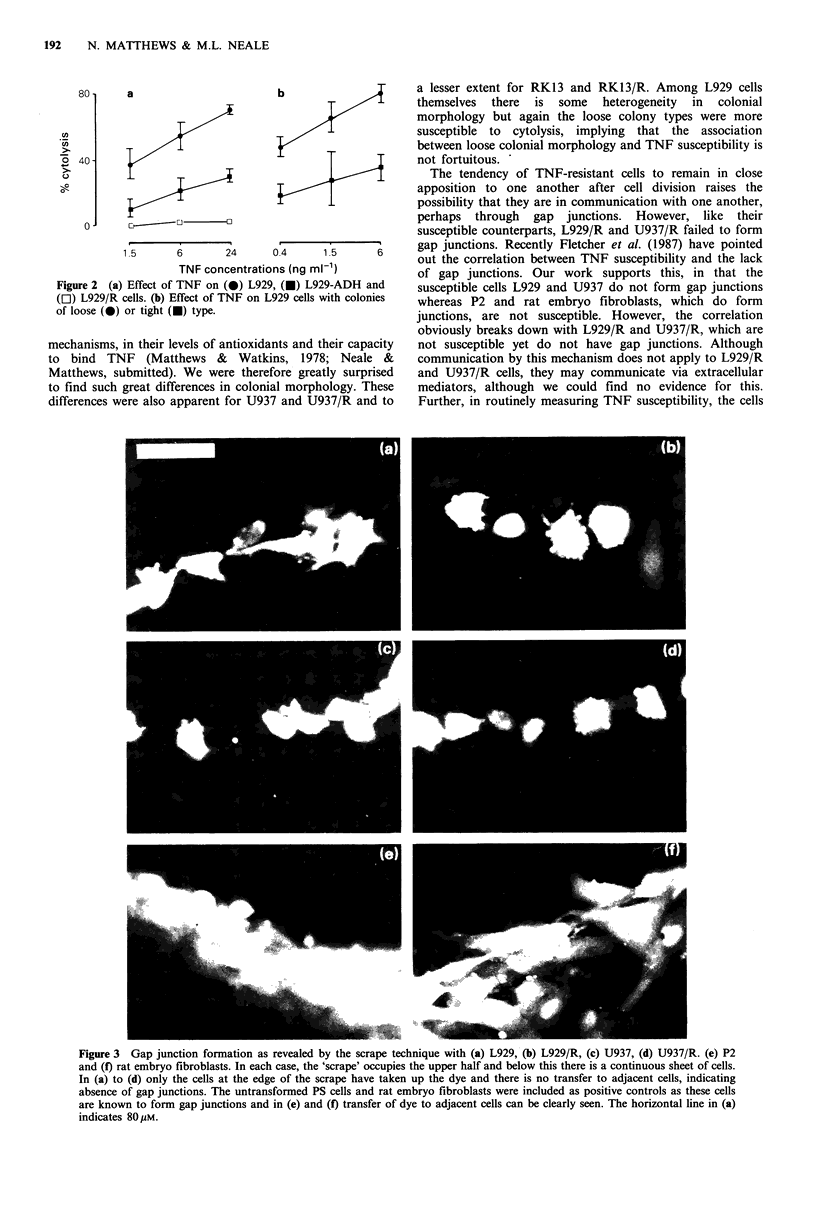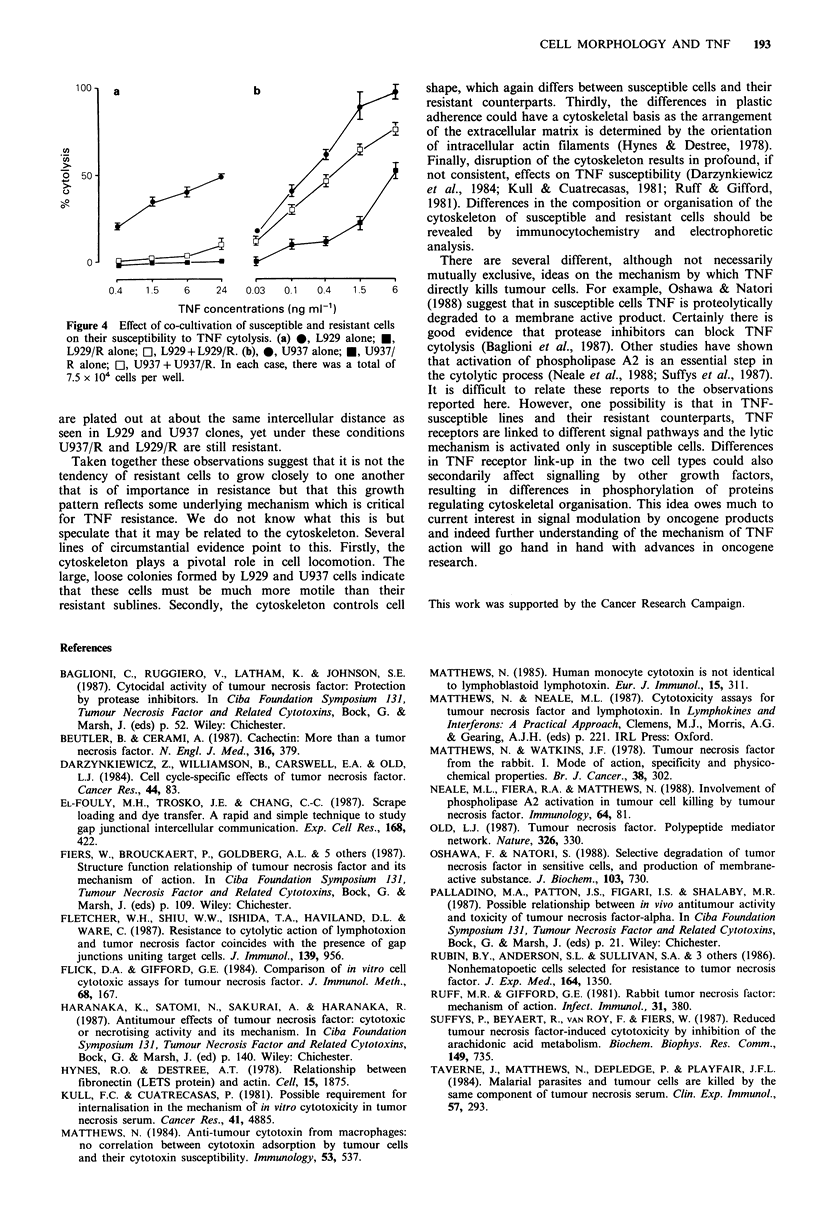# Relationship between tumour cell morphology, gap junctions and susceptibility to cytolysis by tumour necrosis factor.

**DOI:** 10.1038/bjc.1989.39

**Published:** 1989-02

**Authors:** N. Matthews, M. L. Neale

**Affiliations:** Department of Medical Microbiology, University of Wales College of Medicine, Health Park, Cardiff, UK.

## Abstract

**Images:**


Br. J. Cancer (1989), 59, 189–193 © The Macmillan Press Ltd., 1989

### Relationship between tumour cell morphology, gap junctions and susceptibility to cytolysis by tumour necrosis factor

N. Matthews & M.L. Neale

*Department of Medical Microbiology, University of Wales College of Medicine, Heath Park, Cardiff CF4 4XN, UK.*

**Summary** Tumour necrosis factor (TNF) is directly cytolytic to certain tumour cell lines *in vitro*, although TNF-resistant variants can be selected from these susceptible lines by exposure to TNF. While studying TNF-susceptible L929 cells and their resistant variant, L929/R, we noted that within L929 colonies the cells were widely spaced whereas they were closely packed in L929/R colonies. L929/R cells also adhered more strongly to plastic and differed from L929 in cell shape. Similar observations were made with TNF susceptible and resistant variants of two other cell lines (RK13 and a plastic adherent U937 subline). The tendency of resistant cells to grow closely together suggests the possibility of inter-cell communication for the TNF resistant state. However, like L929 and U937, L929/R and U937/R did not communicate by gap junctions and we could find no evidence of extracellular mediators of TNF resistance. Rather the differences in colonial morphology, cell shape and plastic adherence may be secondary to an underlying mechanism which defines TNF susceptibility/resistance.

Tumour necrosis factor (TNF) and the related protein lymphotoxin are produced predominantly by macrophages and lymphocytes respectively. Both proteins have a wide range of biological effects and play key roles in inflammation and immunity (Beutler & Cerami, 1987; Old, 1987) although, as its name suggests, TNF was originally identified on the basis of its activity against tumour cells. *In vivo* the anticancer activity of TNF may be due to direct interaction with tumour cells or indirect, mediated via inflammatory cells and/or tumour endothelium (Fiers *et al.*, 1987; Haranaka *et al.*, 1987; Palladino *et al.*, 1987). *In vitro*, TNF has direct cytocidal or cytostatic effects on certain cell lines and with few exceptions it acts across species. With those cell lines that are susceptible to TNF cytolysis it is relatively easy to select TNF-resistant variants. L929 cells are one of the most susceptible cell lines to *in vitro* cytolysis by TNF and in selecting a TNF-resistant subpopulation we noted morphological differences between the susceptible and resistant variants. This observation prompted the studies described here.

### Materials and methods

### *Materials*

Natural rabbit TNF was prepared as described (Taverne *et al.*, 1984). Human recombinant TNF (rTNF) was kindly provided by Dr G.R. Adolf (Ernst Boehringer Institute, Vienna).

### *Cell lines*

Murine L929 cells and the RK13 cell line of rabbit kidney cells were purchased from Gibco (Paisley, Scotland). U937 cells were originally obtained from Dr M. Greaves (Imperial Cancer Research Fund Labs, London) but an adherent variant which arose spontaneously in this laboratory (Matthews, 1985) was used in this study. All three of these cell lines are susceptible to TNF cytolysis and TNF-resistant variants were selected by culture in natural rabbit TNF (Matthews & Watkins, 1978; Matthews, 1984). The resistant variants have been pulsed regularly with TNF (originally rabbit, latterly human) to prevent reversion to TNF susceptibility. The relative susceptibilities to human TNF of L929, U937 and their variants are shown in Figure 4. RK13 cells are susceptible to rabbit but not human TNF and RK13/R cells require 10–20 times the amount of rabbit TNF for 50% cytolysis in comparison with RK13. P2 is a contact inhibited, untransformed line of rat thyroid fibroblasts and was kindly provided by Dr D. Wynford Thomas (Pathology, University of Wales College of Medicine). This cell line, like primary rat embryo fibroblasts, is not susceptible to TNF cytolysis.

### *Cytolytic assay*

Cells were plated (75 $\mu l$ of $10^5$ $ml^{-1}$) in 96-well microtitre trays and the TNF was added in a 75 $\mu l$ volume. Concentrations given refer to the final culture volume. After 3 days at 37°C the remaining, adherent viable cells were fixed and stained with crystal violet. Dye uptake is proportional to the number of remaining cells and was quantitated photometrically by an ELISA reader. Percentage cytolysis was calculated from the formula $100(a-b)/a$, where $a$ and $b$ are respectively the mean absorbances of triplicate wells without or with TNF. This photometric assay is well established, has been described in detail (Matthews & Neale, 1987) and correlates well with other cytolytic assays (Flick & Gifford, 1984).

### *Gap junctions*

The scrape loading method of El-Fouly *et al.* (1987) was used with minor modifications. Cells were plated 24 h previously in 35 mm Petri dishes in 2 ml volumes at $2 \times 10^5$ $ml^{-1}$. After washing once in isotonic, phosphate-buffered saline (PBS), 1.5 ml Lucifer yellow (Sigma Chemical Co.) was added (0.5 mg $ml^{-1}$ in PBS) and the cell monolayer was scraped lightly with the tip of a micropipette to give three parallel tracks, approx. 7 mm apart. The cells were left for a further 2 min at room temperature and washed five times with PBS. Finally, 2 ml PBS was added and the monolayer was examined by means of a Leitz epifluorescence microscope with a ×25 water immersion objective.

### Results

L929 is a long-established, transformed line of murine fibroblasts which is plastic-adherent, not contact-inhibited and susceptible to TNF cytolysis. Although L929 cells are a heterogeneous population, the majority grow flattened to the plastic and are elongated in appearance. Cells of the TNF-resistant line, L929/R, are less spread and have a more triangular appearance. Growth of L929 cells in L929/R-

Correspondence: N. Matthews.
Received 23 June 1988, and in revised form, 13 September 1988.

conditioned medium does not alter their shape; similarly L929/R cells are unaltered by growth in L929-conditioned medium.

On subculture, it is noticeable that L929/R cells are much more strongly adherent than L929 and require prolonged exposure to trypsin/EDTA to remove all the cells from the surface of culture vessels. Table I summarises an experiment which compares the adherence of L929 and L929/R cells under standardised conditions. An additional, striking difference between L929 and L929/R is their colonial appearance. More than 99% of L929/R colonies are tightly packed with smooth outlines and with the cells in close contact with one another (Figure 1b). L929 colonies are more heterogeneous; the majority are much larger, more loosely packed and with ragged outlines (Figure 1a) although a minority resemble L929/R colonies.

Are the ability to resist trypsin detachment and the capacity to form tight colonies incidental attributes of TNF-resistance or is there some more direct relationship? To test the relationship between plastic adherence and TNF susceptibility, a strongly adherent subpopulation of L929 cells was selected as follows. A confluent culture was exposed to trypsin/EDTA as usual but the majority (>90%) of the cells which detached easily were discarded and the remaining adherent cells were re-fed with medium and allowed to grow to confluency. After four further cycles of selection, the strongly adherent subpopulation of L929 cells (L929/ADH) was tested for TNF susceptibility. As shown in Figure 2a, L929/ADH were more resistant to TNF killing than the parental L929 cells but by no means as resistant as L929/R cells. L929/ADH cells resembled L929/R in their more triangular shape but not in their colonial appearance, which was of the loose variety. However, these observations do suggest that enhanced adherence to plastic does confer some resistance to TNF cytolysis.

To test the possible relationship between colonial appearance and TNF susceptibility, L929 cells were cloned and colonies of the tight and loose varieties were selected

**Table I** Comparison of TNF-susceptible with resistant cell lines for detachment from plastic

| *Experiment no.* | *Cell line* | *% cells after trypsin/EDTA** |
|---|---|---|
| 1 | L929 | 7±2 |
| | L929/R | 31±8 |
| 2 | U937 | 7±3 |
| | U937/R | 55±8 |
| 3 | RK13 | 3±1 |
| | RK13/R | 13±3 |

Cells were cultured overnight in microtitre trays (75 μl amounts of $3 \times 10^5$ ml$^{-1}$, washed once with PBS and triplicate cultures were exposed to either 100 μl PBS or 0.1% trypsin/0.08% EDTA in PBS at room temperature. In each experiment the trypsin/EDTA treated susceptible cells were observed with an inverted microscope, and when the majority had detached the plate was shaken for 10 s on a Dynatech microshaker, the detached cells (susceptible and resistant) were pipetted off and the remaining cells were fixed with formaldehyde and stained with crystal violet. The plate was read on the ELISA reader as for the cytolytic assay and the percentage remaining cells calculated by $100(a/b)$ where $a$, $b$ are the mean absorbance of wells with respectively trypsin/EDTA and PBS. Results are given as the mean ± s.d. within a single representative experiment. There was wide inter-experiment variation in terms of the absolute numbers of cells remaining after trypsinisation. However, in four additional experiments, in every case a significantly higher proportion of resistant cells remained after trypsinisation.

and tested for TNF susceptibility. Eleven well separated colonies were selected, four 'tight' and seven 'loose', and, as shown in Figure 2b, tight clones were much more resistant to TNF killing than loose clones. These results were confirmed in a second independent experiment. Although the tight colony and 'strongly adherent' phenotypes are relatively TNF resistant, they were selected in the absence of TNF, indicating that these phenotypic changes result in increased TNF resistance. We do not know whether the L929/R cells selected from parental L929 cells by exposure to TNF are that subpopulation which has both the tight colony and strongly adherent phenotypes or some other subpopulation.

L929 cells from different sources differ widely in their TNF susceptibility and, even within the same laboratory, L929 cells change in susceptibility with time in the absence of any intentional selection pressure. It may be that this relates to changes in the proportion of cells of tight and loose type. For example, we noted that decreased TNF susceptibility of L929 cells after 3 months of continuous culture was associated with an increase in tight colonies to about 50%.

Other cell lines were also investigated to see whether the phenomena described above are unique to murine L929 cells. A plastic adherent variant of human U937 cells is another TNF-susceptible cell line from which we have selected a TNF-resistant cell line (U937/R). The adherent U937 cells resemble L929 cells in shape but U937/R cells are thinner and more elongated and are unlike L929/R cells. There are also major differences in adherence between the U937 variants (Table I). In terms of colonial appearance U937 is very loose and U937/R more compact (Figure 1c, d). A third TNF susceptible line, rabbit RK13 cells and its resistant variant (RK13/R), was also studied. These lines also differ from one another in appearance, with RK13/R being more flattened to the plastic culture surface; in addition RK13/R adheres more strongly (Table I). Unlike the other two susceptible lines RK13 colonies are relatively tight, although the cells do not appear to be in close contact with one another (Figure 1e): RK13/R colonies are even more compact and the cells appear to be in close contact (Figure 1f).

In all three examples studied the selection of TNF-resistant variants is associated with increased adherence and a more compact colonial morphology. Because resistant cells grow in closer proximity to one another it may be that intercellular communication plays some part in TNF resistance. This could be mediated by products secreted extracellularly or through intracellular mechanisms acting via gap junctions. For example, resistant but not susceptible cells may have functioning gap junctions. This possibility was investigated by means of the recently described scrape technique (El-Fouly *et al.*, 1987). However, although both L929 and U937 cells lacked gap junctions, so did their resistant variants (Figure 3). Since intercellular communication by gap junctions does not explain TNF resistance, the role of extracellular products was examined. In the first set of experiments, conditioned media from resistant cells (L929/R or U937/R) were tested for inhibition of TNF cytolysis of L929 or U937 cells and found to be without effect (data not shown). Because a potential inhibitory factor might have become too diluted in conditioned medium, co-cultivation experiments were performed as follows. Susceptible cells and their resistant variants were mixed together in equal numbers and the mixture was tested for TNF susceptibility. If there is no interaction between the cells then the mixture should exhibit intermediate susceptibility. If the resistant cells are secreting a locally acting inhibitory factor then the mixture should have the TNF resistance of the resistant partner. With U937 and U937/R cells, the mixture was intermediate in susceptibility (Figure 4b), indicating that the resistant cells are not secreting a locally acting TNF inhibitor. With L929 and L929/R cells the mixture did have the TNF resistance of L929/R cells (Figure 4a), indicating that L929/R cells release

**Figure 1** Colonial morphology of (a) L929, (b) L929/R, (c) U937, (d) U937/R, (e) RK13 and (f) RK13/R. Cells were seeded at $10^2$ to $10^3$ per $75\,cm^2$ flask and after 7–9 days were fixed with formaldehyde and stained with crystal violet. The horizontal line in (**a**) indicates $200\,\mu M$ and this scale also applies to (**b**, **c**, **d**, **e** and **f**). (**g**) and (**h**) are taken at lower magnification and represent U937 and U937/R respectively. In (**g**) many of the U937 colonies are too large and diffuse to be seen clearly.

a TNF-inhibitory factor. However, this must be interpreted cautiously, because after 3 days co-cultivation of L929 and L929/R without TNF there were only half as many cells in the mixture as expected and the majority of these resembled L929/R cells. The most likely explanation for this is that the L929/R cells are themselves producing TNF, which eliminates the L929 cells. Indeed, it is well recognised that some resistant L929 derivatives can produce TNF (Rubin *et al.*, 1986). Although we have been unable to detect TNF in L929/R conditioned media enough may be produced to act on neighbouring cells in co-culture. Obviously this phenomenon complicates the interpretation of the experiment and the data cannot be used to support the idea that L929/R cells release a TNF inhibitor.

### Discussion

L929 and L929/R cells are comparable in their susceptibilities to cytotoxic drugs and antibody-dependent killing

**Figure 2** (**a**) Effect of TNF on (●) L929, (■) L929-ADH and (□) L929/R cells. (**b**) Effect of TNF on L929 cells with colonies of loose (●) or tight (■) type.

mechanisms, in their levels of antioxidants and their capacity to bind TNF (Matthews & Watkins, 1978; Neale & Matthews, submitted). We were therefore greatly surprised to find such great differences in colonial morphology. These differences were also apparent for U937 and U937/R and to a lesser extent for RK13 and RK13/R. Among L929 cells themselves there is some heterogeneity in colonial morphology but again the loose colony types were more susceptible to cytolysis, implying that the association between loose colonial morphology and TNF susceptibility is not fortuitous.

The tendency of TNF-resistant cells to remain in close apposition to one another after cell division raises the possibility that they are in communication with one another, perhaps through gap junctions. However, like their susceptible counterparts, L929/R and U937/R failed to form gap junctions. Recently Fletcher *et al.* (1987) have pointed out the correlation between TNF susceptibility and the lack of gap junctions. Our work supports this, in that the susceptible cells L929 and U937 do not form gap junctions whereas P2 and rat embryo fibroblasts, which do form junctions, are not susceptible. However, the correlation obviously breaks down with L929/R and U937/R, which are not susceptible yet do not have gap junctions. Although communication by this mechanism does not apply to L929/R and U937/R cells, they may communicate via extracellular mediators, although we could find no evidence for this. Further, in routinely measuring TNF susceptibility, the cells

**Figure 3** Gap junction formation as revealed by the scrape technique with (**a**) L929, (**b**) L929/R, (**c**) U937, (**d**) U937/R. (**e**) P2 and (**f**) rat embryo fibroblasts. In each case, the 'scrape' occupies the upper half and below this there is a continuous sheet of cells. In (**a**) to (**d**) only the cells at the edge of the scrape have taken up the dye and there is no transfer to adjacent cells, indicating absence of gap junctions. The untransformed PS cells and rat embryo fibroblasts were included as positive controls as these cells are known to form gap junctions and in (**e**) and (**f**) transfer of dye to adjacent cells can be clearly seen. The horizontal line in (**a**) indicates 80 $\mu$M.

**Figure 4** Effect of co-cultivation of susceptible and resistant cells on their susceptibility to TNF cytolysis. (**a**) ●, L929 alone; ■, L929/R alone; □, L929 + L929/R. (**b**), ●, U937 alone; ■, U937/R alone; □, U937 + U937/R. In each case, there was a total of $7.5 \times 10^4$ cells per well.

are plated out at about the same intercellular distance as seen in L929 and U937 clones, yet under these conditions U937/R and L929/R are still resistant.

Taken together these observations suggest that it is not the tendency of resistant cells to grow closely to one another that is of importance in resistance but that this growth pattern reflects some underlying mechanism which is critical for TNF resistance. We do not know what this is but speculate that it may be related to the cytoskeleton. Several lines of circumstantial evidence point to this. Firstly, the cytoskeleton plays a pivotal role in cell locomotion. The large, loose colonies formed by L929 and U937 cells indicate that these cells must be much more motile than their resistant sublines. Secondly, the cytoskeleton controls cell shape, which again differs between susceptible cells and their resistant counterparts. Thirdly, the differences in plastic adherence could have a cytoskeletal basis as the arrangement of the extracellular matrix is determined by the orientation of intracellular actin filaments (Hynes & Destree, 1978). Finally, disruption of the cytoskeleton results in profound, if not consistent, effects on TNF susceptibility (Darzynkiewicz *et al.*, 1984; Kull & Cuatrecasas, 1981; Ruff & Gifford, 1981). Differences in the composition or organisation of the cytoskeleton of susceptible and resistant cells should be revealed by immunocytochemistry and electrophoretic analysis.

There are several different, although not necessarily mutually exclusive, ideas on the mechanism by which TNF directly kills tumour cells. For example, Oshawa & Natori (1988) suggest that in susceptible cells TNF is proteolytically degraded to a membrane active product. Certainly there is good evidence that protease inhibitors can block TNF cytolysis (Baglioni *et al.*, 1987). Other studies have shown that activation of phospholipase A2 is an essential step in the cytolytic process (Neale *et al.*, 1988; Suffys *et al.*, 1987). It is difficult to relate these reports to the observations reported here. However, one possibility is that in TNF-susceptible lines and their resistant counterparts, TNF receptors are linked to different signal pathways and the lytic mechanism is activated only in susceptible cells. Differences in TNF receptor link-up in the two cell types could also secondarily affect signalling by other growth factors, resulting in differences in phosphorylation of proteins regulating cytoskeletal organisation. This idea owes much to current interest in signal modulation by oncogene products and indeed further understanding of the mechanism of TNF action will go hand in hand with advances in oncogene research.

This work was supported by the Cancer Research Campaign.

### References

BAGLIONI, C., RUGGIERO, V., LATHAM, K. & JOHNSON, S.E. (1987). Cytocidal activity of tumour necrosis factor: Protection by protease inhibitors. In *Ciba Foundation Symposium 131, Tumour Necrosis Factor and Related Cytotoxins*, Bock, G. & Marsh, J. (eds) p. 52. Wiley: Chichester.

BEUTLER, B. & CERAMI, A. (1987). Cachectin: More than a tumor necrosis factor. *N. Engl. J. Med.*, **316**, 379.

DARZYNKIEWICZ, Z., WILLIAMSON, B., CARSWELL, E.A. & OLD, L.J. (1984). Cell cycle-specific effects of tumor necrosis factor. *Cancer Res.*, **44**, 83.

EL-FOULY, M.H., TROSKO, J.E. & CHANG, C.-C. (1987). Scrape loading and dye transfer. A rapid and simple technique to study gap junctional intercellular communication. *Exp. Cell Res.*, **168**, 422.

FIERS, W., BROUCKAERT, P., GOLDBERG, A.L. & 5 others (1987). Structure function relationship of tumour necrosis factor and its mechanism of action. In *Ciba Foundation Symposium 131, Tumour Necrosis Factor and Related Cytotoxins*, Bock, G. & Marsh, J. (eds) p. 109. Wiley: Chichester.

FLETCHER, W.H., SHIU, W.W., ISHIDA, T.A., HAVILAND, D.L. & WARE, C. (1987). Resistance to cytolytic action of lymphotoxion and tumor necrosis factor coincides with the presence of gap junctions uniting target cells. *J. Immunol.*, **139**, 956.

FLICK, D.A. & GIFFORD, G.E. (1984). Comparison of *in vitro* cell cytotoxic assays for tumour necrosis factor. *J. Immunol. Meth.*, **68**, 167.

HARANAKA, K., SATOMI, N., SAKURAI, A. & HARANAKA, R. (1987). Antitumour effects of tumour necrosis factor: cytotoxic or necrotising activity and its mechanism. In *Ciba Foundation Symposium 131, Tumour Necrosis Factor and Related Cytotoxins*, Bock, G. & Marsh, J. (ed) p. 140. Wiley: Chichester.

HYNES, R.O. & DESTREE, A.T. (1978). Relationship between fibronectin (LETS protein) and actin. *Cell*, **15**, 1875.

KULL, F.C. & CUATRECASAS, P. (1981). Possible requirement for internalisation in the mechanism of *in vitro* cytotoxicity in tumor necrosis serum. *Cancer Res.*, **41**, 4885.

MATTHEWS, N. (1984). Anti-tumour cytotoxin from macrophages: no correlation between cytotoxin adsorption by tumour cells and their cytotoxin susceptibility. *Immunology*, **53**, 537.

MATTHEWS, N. (1985). Human monocyte cytotoxin is not identical to lymphoblastoid lymphotoxin. *Eur. J. Immunol.*, **15**, 311.

MATTHEWS, N. & NEALE, M.L. (1987). Cytotoxicity assays for tumour necrosis factor and lymphotoxin. In *Lymphokines and Interferons: A Practical Approach*, Clemens, M.J., Morris, A.G. & Gearing, A.J.H. (eds) p. 221. IRL Press: Oxford.

MATTHEWS, N. & WATKINS, J.F. (1978). Tumour necrosis factor from the rabbit. I. Mode of action, specificity and physico-chemical properties. *Br. J. Cancer.*, **38**, 302.

NEALE, M.L., FIERA, R.A. & MATTHEWS, N. (1988). Involvement of phospholipase A2 activation in tumour cell killing by tumour necrosis factor. *Immunology*, **64**, 81.

OLD, L.J. (1987). Tumour necrosis factor. Polypeptide mediator network. *Nature*, **326**, 330.

OSHAWA, F. & NATORI, S. (1988). Selective degradation of tumor necrosis factor in sensitive cells, and production of membrane-active substance. *J. Biochem.*, **103**, 730.

PALLADINO, M.A., PATTON, J.S., FIGARI, I.S. & SHALABY, M.R. (1987). Possible relationship between *in vivo* antitumour activity and toxicity of tumour necrosis factor-alpha. In *Ciba Foundation Symposium 131, Tumour Necrosis Factor and Related Cytotoxins*, Bock, G. & Marsh, J. (eds) p. 21. Wiley: Chichester.

RUBIN, B.Y., ANDERSON, S.L. & SULLIVAN, S.A. & 3 others (1986). Nonhematopoetic cells selected for resistance to tumor necrosis factor. *J. Exp. Med.*, **164**, 1350.

RUFF, M.R. & GIFFORD, G.E. (1981). Rabbit tumor necrosis factor: mechanism of action. *Infect. Immunol.*, **31**, 380.

SUFFYS, P., BEYAERT, R., VAN ROY, F. & FIERS, W. (1987). Reduced tumour necrosis factor-induced cytotoxicity by inhibition of the arachidonic acid metabolism. *Biochem. Biophys. Res. Comm.*, **149**, 735.

TAVERNE, J., MATTHEWS, N., DEPLEDGE, P. & PLAYFAIR, J.F.L. (1984). Malarial parasites and tumour cells are killed by the same component of tumour necrosis serum. *Clin. Exp. Immunol.*, **57**, 293.